# American Pharmaceutical Association

**Published:** 1876-11

**Authors:** 


### AMERICAN PHARMACEUTICAL ASSOCIATION.

The twenty-fourth annual meeting of the Association commenced at the College of Pharmacy, Philadelphia, September 12th, 1876, and at half-past three o'clock P.M., was called to order by its President, Prof. George F. H. Markoe, of Boston. The chair appointed William Neergaard, of New York; W. J. M. Gordon, of Cincinnati; and J. P. Remington, of Philadelphia, a Committee on Credentials. * * * * *

The Committee on Nominations presented their report, recommending for officers for the ensuing year:

*President*—Charles Bullock, of Philadelphia.

*Vice-Presidents*—S. A. D. Sheppard, Boston; Geo. J. Luhn, Charleston, S. C.; J. D. Wells, Cincinnati, O.

*Treasurer*—C. A. Tufts, Dover, N. H.

*Permanent Secretary*—Prof. J. M. Maisch, Philadelphia.

*Reporter on Pharmacy*—Prof. C. Lewis Diehl, Louisville, Kentucky.

*Local Secretary*—Henry J. Rose, Toronto, Canada.

*Executive Committee*—George W. Kennedy, Pottsville, Pa.; C. H. Dalrymple, W. H. Crawford, John Ingalls, Prof. J. M. Maisch.

*Committee on Drug Market*—William Saunders, London, Ontario; W. H. Wickham, Professor J. F. Judge, Prof. N. G. Bartlett, Charles F. G. Meyer.

*Committee on Papers and Queries*—William McIntyre, Philadelphia; Louis J. Dohme, Joseph L. Lemberger.

*Business Committee*—Joseph Roberts, Baltimore, Md.; H. D. Wellcome, Charles Rice. * * * * * *

The Committee on Legislation reported, through Professor Maisch, that no great changes had occurred in this department during the past year. In two States the law had been amended. In Baltimore the law permits the sale of patent and proprietary medicines by any one, as also the sale of medicine put up in bottles or packages by wholesale druggists. In South Carolina the examinations were formerly conducted by the medical colleges, but now the board is constituted by four pharmaceutists and two physicians, and the receipts of the board are divided equally between the Pharmaceutical Association and the medical colleges. No other changes of note were alluded to in the report, which was then adopted. * * *

The report on Metrical Weights and Measures was read by Dr. F. Hoffman. The system was reported feasible, and the committee recommended that it be adopted for all wants, and that our Government should fix a time after which it should be the legal standard of the country.

Mr. T. S. Wiegand, one of the committee, read a short note stating his reasons for signing the report; also giving other reasons why he was not in full accord with all its conclusions.

On motion, both reports were directed to be published in the proceedings. * * * * * *

Dr. E. R. Squbb was requested by the Committee on Papers and Queries to read a volunteer paper on Phosphorus, which he proceeded to do.

After the reading of the paper, Dr. Squibb offered the following resolution:

*Resolved*, That the American Pharmaceutical Association devote an hour of its third session to a discussion of its interests in the United States Pharmacopœia, with a view to the adoption or rejection of the following resolution:

WHEREAS, By action of the American Medical Association at its recent meeting in this city, it is proposed to discuss at its next meeting in Detroit, in June, 1877, a proposition for that Association to assume the control of the National Pharmacopœia; therefore,

*Resolved*, That this Association offers to the American Medical Association its hearty co-operation in the work in any way that the American Medical Association may find the services of this Association most useful.

*Resolved*, That a copy of this preamble and resolutions, with the discussion had thereupon, be forwarded by the President of this Association to the President of the American Medical Association. * * * * * * *

The committee to select a time and place for the next meeting, reported that they recommend it be held in Toronto, Ontario, September 4th, 1877, at 3 o'clock P. M.

Mr. Shoemaker read selections from the report of the Committee on Drug Market.

The report alluded to the changes in values, amount and character of many of the articles used in the wholesale trade.

It was stated that the total imports for the fiscal year were $476,000,000, while the total exports were $596,000,000, leaving a balance in our favor of $20,000,000.

The character of many of the powdered drugs of the market was alluded to, and their quality hinted at by the comparative prices at which they were sold, and the value of good crude drugs. Borax was exported to the extent of 3,000,000 pounds, while California and Nevada have produced in all, during the year, 6,000,000.

The Pacific Coast has furnished during the year 54,000 flasks of mercury, while less than that has been produced by all the other nations of the world combined. The product of opium was stated to be smaller in amount for the year than was anticipated, and it would probably advance in price still further. Many other interesting subjects were touched upon. The report concluded with a list of imported drugs for the past fiscal year. It was accepted and referred for publication.

QUERY 8. Five grains of citric acid added to the finished product of the formula of the U. S. P. for syrup of ferrous iodide, seems to preserve *unchanged* the appearance of the syrup. Is this addition admissible?—DR. WILSON R. PILE, Philadelphia.

The experiments of Dr. Wilson H. Pile, in answer, failed to satisfy him as to the value of citric acid as a preservative for this changeable syrup, and the paper was chiefly a negative result.

Discussion which followed gave more favorable results as to its tendency to protect this syrup from its proneness to change. * * * * * *

QUERY 11. The fluid extract of cotton-root bark occasionally gelatinizes on keeping. Can a modification of the process for making this fluid extract be suggested which will prevent such a change? To which principle, and to what influences is it due, and has the bark of the green root any superiority over that of the dry in the preparation of the fluid extract; by J. M. LLOYD, Cincinnati, Ohio.

The writer details many very careful experiments in the manufacture of fluid extract of cotton-root bark, both dry and fresh. He gives preference to an alcoholic menstruum, but obtained satisfactory results in making an extract which, in his experience, *never gelatinized,* when the menstruum used was alcohol ten parts (fluid) to glycerine six parts (fluid). Numerous trials by physicians indicate plainly the *unreliability* of any preparation made from the dried cotton bark. On the other hand, when the *fresh* bark was used, and a fluid extract made from this was tried carefully by a large number of physicians, the almost universal report was favorable, the action being to facilitate parturition; and in other cases, where it was sought to re-establish suppressed menstruation, it scarcely ever failed. His conclusions, therefore, point clearly to the use of the *fresh bark only.* . . . . . . . . .

QUERY 29. Is it advisable to have an officinal aromatic spirit that will represent a fine refreshing cologne, to be used in lotions and for the sick room? If so, give a formula and name for the preparation. By GEORGE LEIS, Lawrence, Kansas.

The writer came to the conclusion that any aromatic spirit, to be grateful in the sick room, must be devoid of odors that are too sweet, and thinks that a plain odor is more lasting in its usefulness for the sick room. He therefore proposes a spirit in which bergamot, cassia and lavender are the odors. In connection with its intended use in the sick room, he deemed it absolutely necessary that it should contain some ingredient which should act as a deodorizer and preventive of foul odors. This would indicate an addition of some of the well known disinfectants or deodorizers, and he thinks the addition of salicylic acid accomplishes the purpose. The formula contributed included, therefore, this acid to the extent of ten grains to the ounce of the perfume given. The name proposed was "Lotio Antiseptica Fragrans," which would, he thought, sufficiently indicate its character and uses. The best results were to be obtained by freely using it as a "spray" in the sick room.

QUERY 30. What advantages would result from the substitution of parts by weight for absolute quantities in the revision of the Pharmacopœia, and if any disadvantages other than those incident to change, what are they? By By PROF. S. P. SHARPLES, Boston.

The writer objects *in toto* to the use of parts by weight, on the ground that it is neither convenient nor practicable. He instances a few preparations which it would seem almost impossible to bring to such proportions as would make it manageable or meet the exigences of the average apothecary. The additional objection is made to the trouble of frequent weighing of many of the fluids in use as being more tedious and not as accurate for common use as that of measuring.

Considerable discussion followed this paper, but several expressed themselves as favoring the change.

QUERY 31. What advantage is there in retaining alcohol in tinctura ferri chloridi? Would it be desirable to introduce liquor ferri chlor. dilut. of the same iron strength in place of the tincture? By M. S. BIDWELL, Elmira, N. Y.

The writer concludes from his investigations that a watery solution would not be as acceptable to the medical faculty; that it would take too much trouble and time to introduce; but favors the reduction of the alcoholic strength to correspond to dilute alcohol.

QUERY 32. What is lacto-peptin? By EMIL SCHEFFER, Louisville, Ky.

The writer's careful experiments are detailed in full in his paper in reply to this question. It will be sufficient to say that taking the published formula as stated on the label, and trying the experiments suggested by the makers, they fail to prove or produce the results claimed. The digestive power of the powder proves the presence of pepsin, and as coagulated albumen is dissolved by lacto-peptin, the presence of pepsin is confirmed. Pancreatin in dilute alkaline solution, when added to a small quantity of neutral lard, will produce acidification. Pancreatin is coagulated by heat, but neither of the above results are found when lacto-peptin is tested in a suitable manner. When starch paste was used, it was not converted into sugar by lacto-peptin, which fails to provê the presence of diatase. The careful experiments of Mr. Scheffer prove the presence of pepsin, sugar, lactic acid, hydrochloric acid, starch, cellular tissue, mucus, and fat, but *no pancreatin nor diatase*, and that it is inferior in digestive strength to the saccharated pepsin of the market. . . . . . .

QUERY 39. How much acacia is needed to emulsify perfectly the fixed or volatile oils and balsams? By E. GREGORY, Lindsay, Ontario.

Mr. Gregory gives several detailed processes of manipulation, as laid down in the various works that come under notice of pharmaceutists. His judgment is that three parts of acacia are needed to readily emulsify volatile and fixed oils and balsams, and that mucilage which is kept already made is not so good as that made freshly for the purpose. The process of manipulation preferred is detailed in *The Druggists' Circular*, December, 1874, in which powdered acacia, one part, is mixed with two parts oil, and water, one and one-half parts, afterwards at once. Numerous other experiments are given, but this in his hands has proved most rapid, the whitest, the oil globules most readily divided, and the emulsion to be longest retained in intimate mixture without separation.—*Druggists' Circular.*

